# Remote Sensing Derived Fire Frequency, Soil Moisture and Ecosystem Productivity Explain Regional Movements in Emu over Australia

**DOI:** 10.1371/journal.pone.0147285

**Published:** 2016-01-22

**Authors:** Nima Madani, John S. Kimball, Mona Nazeri, Lalit Kumar, David L. R. Affleck

**Affiliations:** 1 Numerical Terradynamic Simulation Group, College of Forestry & Conservation, University of Montana, 32 Campus Drive Missoula, MT, 59812, United States of America; 2 School of Journalism, Department of Environmental Journalism, University of Montana, 32 Campus Drive, Missoula, MT, 59812, United States of America; 3 Ecosystem Management, School of Environmental and Rural Science, University of New England, Ring Road, Armidale, New South Wales, 2351, Australia; 4 College of Forestry and Conservation, University of Montana, 32 Campus Drive, Missoula, MT, 59812, United States of America; University of Calgary, CANADA

## Abstract

Species distribution modeling has been widely used in studying habitat relationships and for conservation purposes. However, neglecting ecological knowledge about species, e.g. their seasonal movements, and ignoring the proper environmental factors that can explain key elements for species survival (shelter, food and water) increase model uncertainty. This study exemplifies how these ecological gaps in species distribution modeling can be addressed by modeling the distribution of the emu (*Dromaius novaehollandiae*) in Australia. Emus cover a large area during the austral winter. However, their habitat shrinks during the summer months. We show evidence of emu summer habitat shrinkage due to higher fire frequency, and low water and food availability in northern regions. Our findings indicate that emus prefer areas with higher vegetation productivity and low fire recurrence, while their distribution is linked to an optimal intermediate (~0.12 m^3^ m^-3^) soil moisture range. We propose that the application of three geospatial data products derived from satellite remote sensing, namely fire frequency, ecosystem productivity, and soil water content, provides an effective representation of emu general habitat requirements, and substantially improves species distribution modeling and representation of the species’ ecological habitat niche across Australia.

## Introduction

A variety of satellite remote sensing derived global environmental data products have been available for decades, with many offering observations suitable for a range of ecological applications [1]. Among these observational data records, vegetation greenness indices, including the Normalized Difference Vegetation Index (NDVI) and enhanced vegetation index (EVI), provide an effective proxy for vegetation cover, terrestrial productivity and food availability, and have been widely used to define species habitat relationships and for species distribution modelling [2–4]. The species distribution modelling framework based on Hutchinson’s (1957) fundamental niche theory has been widely used to predict core species habitats and critical requirements for survival by linking species presence-absence data with a set of explanatory environmental variables. In these statistical models, the ecological knowledge about the species and their habitat is critical for species distribution modeling [5,6]. In this regard, satellite remote sensing observations can be used to fill the ecological gaps in species distribution modelling by providing spatially continuous observations and environmental proxies for the three basic criteria governing species survival: shelter, food and water, and their relative spatial and temporal distributions.

Disturbance-related observations such as fire frequency can provide information about shelter extent and quality influencing species distribution and survival, whereby areas with recent disturbance or high fire frequency may have less suitable cover than other areas with lower fire disturbance levels. However, wildfire is not always a limiting distribution factor and may even promote species presence or abundance by enhancing available forage. In North America for example, moose populations increase in early plant successional stages following fire disturbance [7]. Fire as a historical event [8] is interactive with climate and plays an important role in ecosystem dynamics. Fire regimes in the landscape can affect climate [9,10], plant communities [11], food availability [12], and species abundance and distribution [13,14]. Therefore, knowledge of species response to fire disturbance is critical for ecological management [15]. Australia is one of the most wildfire prone areas on the planet [16], and the associated fire disturbance and recovery regimes exert a strong influence on regional habitat distributions and migration patterns for many bird species [14]. However, only a limited number of studies in Australia have explored the effect of fire and seasonal climate variations on bird distributions (e.g. [14,17–19]).

Ecosystem productivity is directly linked to food availability for species [20]. Even though vegetation greenness indices, such as the NDVI, are correlated with photosynthetic canopy cover and vegetation productivity, the total productivity varies across the landscape based on vegetation morphological characteristics and environmental conditions. Remote sensing based vegetation productivity estimates can provide useful information regarding potential changes in food availability for herbivores and can respond to environmental change [21,22].

Soil moisture and rainfall data can provide information regarding water availability for animals. Global spatially gridded rainfall data are available from the WorldClim database [23] and have been extensively used for ecological studies (e.g. [3,22,23]). On the other hand, satellite remote sensing based surface (<5 cm depth) soil moisture data records are available spanning multiple decades (from 1978) as derived by multiple overlapping active and passive microwave sensors [24–26]. These data provide consistent global information relating to potential water availability for species that may have utility for modeling species distribution and associated niche space.

In this study, we applied a set of global satellite remote sensing data records including the Global Fire Emissions Database (GFED4) [27], the MOD17 GPP product [21], and a global surface soil moisture data record [26,28,29] as proxies for critical species habitat requirements within an empirical modeling framework to explain movement patterns for the emu (*Dromaius novaehollandiae*), a large native flightless bird species of Australia. Emus can be found over a wide range of habitats and exhibit a large seasonal movement range across Australia [30]. We explored the effect of fire frequency and food and water availability on emu presence, and also tested the effectiveness of other satellite environmental data records as alternative metrics representing food and water availability, including MODIS (MOD13C1 CMG) NDVI [31], and Tropical Rainfall Measuring Mission (TRMM) rainfall data [32].

## Methods

### Species Data

**Fig 1:**
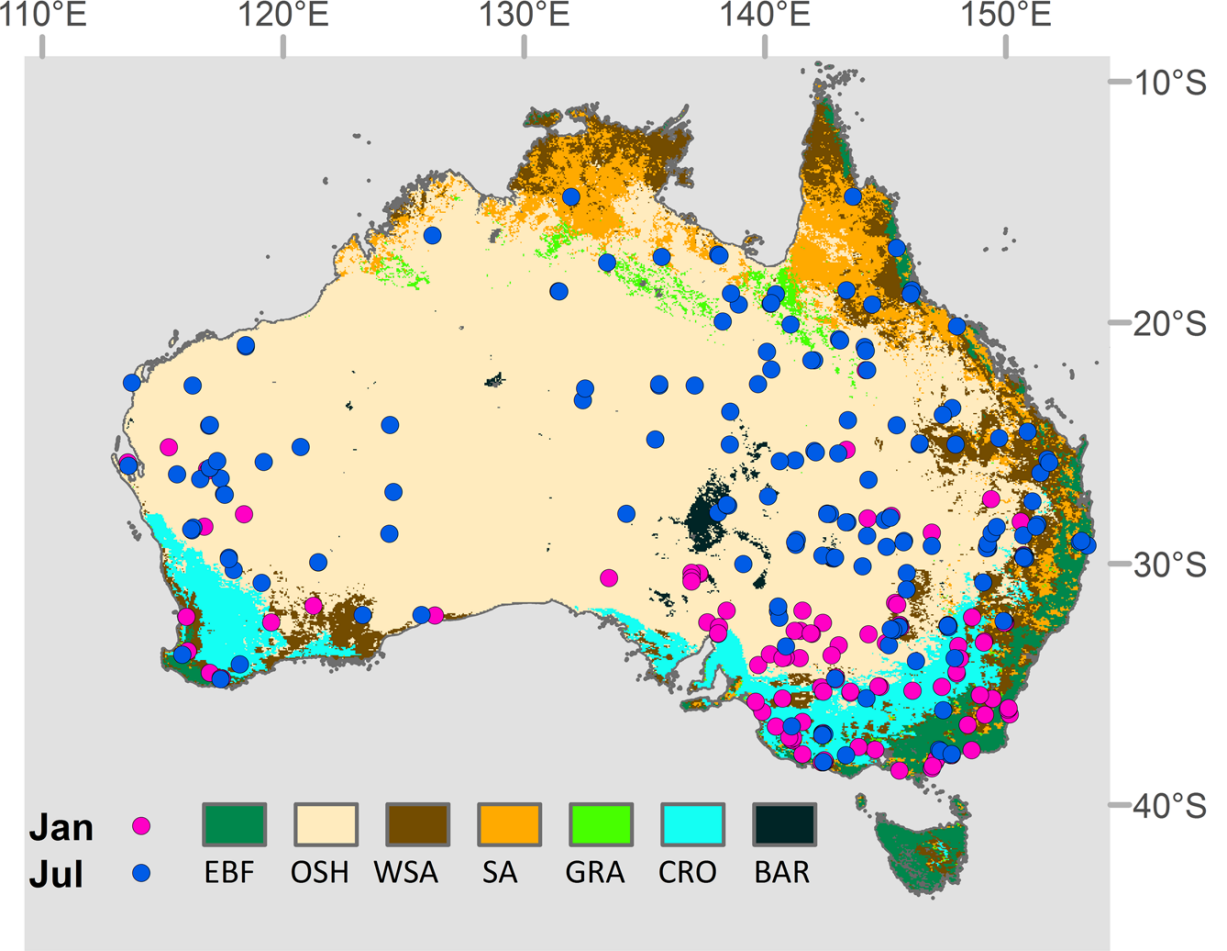
The presence record of emu (*Dromaius novaehollandiae*) in January (summer) and July (winter) in Australia reported from the Atlas of Australian Birds (Barrett et al., 2003) superimposed on a MODIS MOD12Q1-UMD global land cover map [38] where the following vegetation classes each cover more than one percent of the total area: EBF (Evergreen Broadleaf Forest), OSH (Open Shrubland), WSA (Woody Savanna), SA (Savanna), GRA (Grassland), CRO (Cropland), and BAR (Barren).

The emu has been categorized as a species of least concern on the International Union for Conservation of Nature **(**IUCN) red list [33]. Emus occupy a wide range of habitats in Australia and can be found in savannas, grasslands and subtropical regions [34]. Emus also exhibit a regionally extensive seasonal movement pattern that is reported to be a function of rainfall patterns [30]. Because of their broad movement range, emus play a significant role in the seed dispersal of many Australian plant species [35,36]. We used 240 summer and winter species occurrence records from the New Atlas of Australian Birds [37], where monthly spatial observation records from birdwatchers are available from 1998. The associated emu presence data indicate that the geographic distribution of this species shrinks during the austral summer (January) and increases to its broadest range in the austral winter (July) (Fig 1).

In order to investigate potential environmental factors influencing summer habitat reduction, 120 presence data points for the month of January were used for further analysis. Because emus have large daily movement patters [39], pseudo-absence data were randomly sampled from outside of a 10 km radius window around each presence point to determine the relationship between emu presence and associated habitat conditions defined from the satellite observational records.

### Explanatory Variables

Explanatory variables were created to represent emu summer habitat characteristics (21 June—21 September) from 2000–2010. We used GFED4 monthly burned area data with 0.25 degree spatial resolution [27] to create fire frequency data. The GFED4 data are created using ensemble satellite observations including MODIS (MCD 64A1) burned area, Tropical Rainfall Measuring Mission (TRMM) and Visible and Infrared Scanner (VIRS) monthly active fire observations, and Along-Track Scanning Radiometer (ATSR) data. The fire frequency data show the number of times a pixel has been burned within the observational record, extending from 2000–2010 for this study. The resulting fire frequency values range from 0 (no recorded fire record) to 11 years, representing the highest fire frequency for the study record.

The Moderate Resolution Image Spectroradiometer (MODIS) MOD17 Gross Primary Productivity (GPP) product [21,40] is based on the light use efficiency (LUE) concept [41], in which plant production is linearly related to photosynthetically active radiation (PAR) absorbed by the vegetation canopy (APAR), and the efficiency with which this solar radiant energy is transformed into vegetation biomass, which varies according to plant functional type and climate variability across ecosystems [42]. The MODIS MOD17 global operational GPP data record extends from 2000 to present with 8-day temporal fidelity and 1-km spatial resolution compatible with MODIS global geographic projection tiles. However, here we used the global mosaicked images with 0.05 degree spatial resolution (~ 5 km) from the MODIS MOD17 A2 C5 product. The MOD17 product is created using MODIS derived fractional photosynthetic active radiation (FPAR) [43] in a light use efficiency modeling framework. The 8-day GPP data were disaggregated to a daily time step, and the average GPP for Austral winter and summer months was determined from the 2000–2010 record.

The global soil moisture data record used for this study extends from November 1978 to December 2010 and was created using merged and calibrated overlapping satellite active and passive microwave sensor observations including SMMR, SSM/I, TMI, ASMR-E, AMI-WS, and ASCAT [26,28,29]. The surface (<5cm depth) soil moisture record is available at a daily time step and 0.25 degree spatial resolution in volumetric (m^3^ m^-3^) units. We used the data set to derive average austral winter (JJA) and summer (DJF) soil moisture levels over the 2000–2010 record for Australia. For the analysis, we only used the highest quality soil moisture data with consistent agreement among all of the component sensor retrievals.

**Fig 2:**
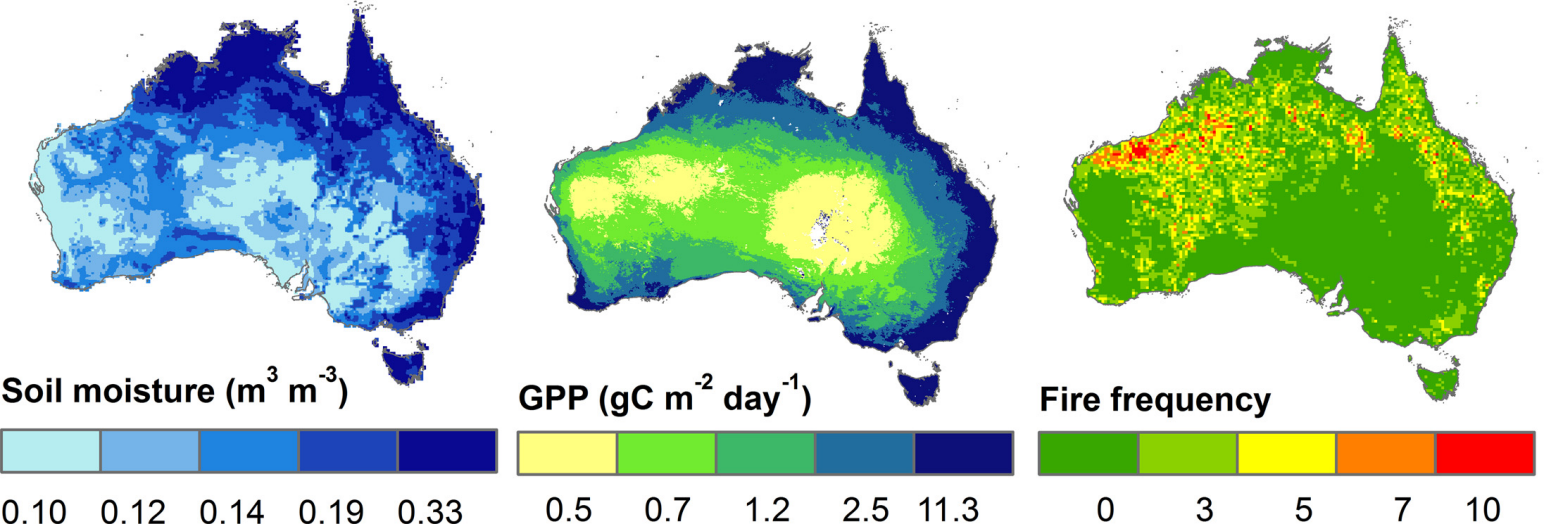
Satellite remote sensing derived environmental variables used to explain emu summer habitat suitability, including soil moisture, gross primary production (GPP) and fire frequency used as respective habitat suitability metrics for water availability, food supply and shelter conditions.

For spatial consistency, we resampled the fire frequency and soil moisture data to the same 0.05 degree spatial resolution (geographic projection) as the MODIS GPP data record using a nearest-neighbor resampling technique (Fig 2).

The average austral summer NDVI was also derived over the domain using the MODIS (MOD13C1 CMG) global monthly, 0.05 degree spatial resolution NDVI product for the 2000–2010 record [31]. TRMM rainfall data were acquired at 3 hourly intervals and 0.25 degree spatial resolution over the 11 year study record, and used to estimate mean austral summer precipitation over the domain. The rainfall data were resampled to a 0.05 degree spatial resolution and geographic projection consistent with the other variables. Rainfall data processing was conducted using Google Earth Engine (GEE) [44,45], which provides a web based platform where multiple data sets can be acquired and analyzed in an efficient way using the Google data servers.

### Statistical Analysis

Environmental variables for predicting emu summer distribution were selected based on the amount of variance explained and lowest Akaike information criterion (AIC) after checking for co-linearity. Co-linearity in logistic regression models can lead to unreliable estimates of the model coefficients. In this study, variables having less than 70% Pearson correlation (Table A in S1 File) were considered for generalized additive models (GAM) [46] implemented in the R programing environment [47]. We used the GAM with binomial family distribution and logit link to estimate emu seasonal habitat pattern shifts between winter and summer based on the survey presence location observations. The difference between the generalized linear model and GAM approach is that the GAM adds smoothed non-parametric functions (here thin plate regression splines) to the parametric part of the generalized linear model (GLM), and in this regard improves GLM performance [48]. We used linear functions for all the variables except soil moisture and rainfall.

The habitat suitability model performance was assessed using a threshold independent measure of the Area Under the Curve (AUC) of the Receiver Operator Characteristic (ROC) plot using the ROCR library [49] in R. The AUC is a dimensionless metric that varies between 0 and 1, where values close to 1 represent greater model accuracy. We used a 10-fold cross validation technique, setting aside 20% of the data for validation and estimating model parameters with the remaining 80% of the observations. We repeated this procedure 10 times and derived the ensemble mean of the predicted suitability map, and the average AUC of the test data points. We classified the predictions based on the overall performance indicated by the rate of true positive versus false positive predictions.

## Results

The best GAM in explaining emu spatial distributions over the domain resulted from using fire frequency, soil moisture and GPP as predictor variables. The resulting GAM showed an adjusted R^2^ of 68.5% (*p* < 0.0001) and AIC of 122 with an average AUC of 98% (Fig A in S1 File). An alternative GAM using TRMM rainfall and NDVI instead of soil moisture and GPP resulted in slightly lower model R^2^ performance (64%) and higher AIC of 126 (Fig B in S1 File).

**Fig 3:**
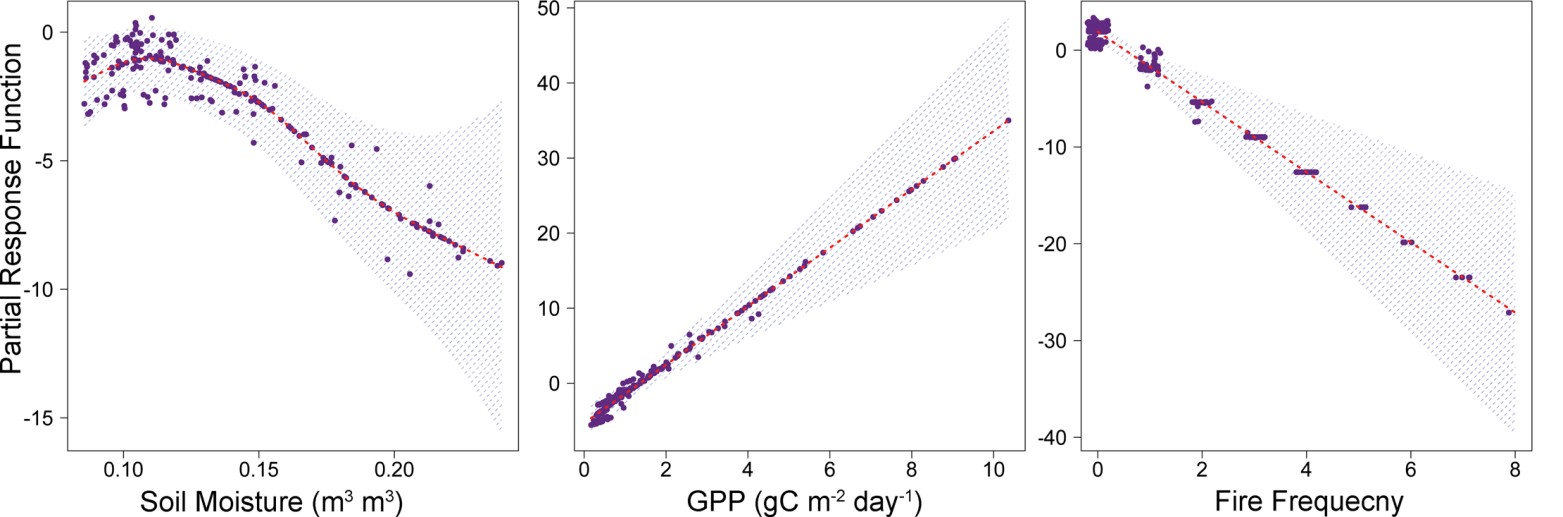
Partial response and residual plots from the fitted GAM using soil moisture, GPP, and fire frequency as explanatory variables for predicting emu habitat distribution; shading denotes the 95% confidence interval associated with the model estimates, and partial residuals of the presence/pseudo-absence observations are shown as points.

In the fitted model, soil moisture has a nonlinear relationship with the response variable on the logit scale such that soil water content has a positive relationship with emu presence up to an intermediate level of approximately 0.12 m^3^ m^-3^, while larger soil moisture values are associated with reduced emu presence and degraded habitat suitability (Fig 3). These results indicate that emus occupy a zone of optimal summer habitat conditions between relatively wet and dry regions.

As expected, GPP used as a proxy for food availability exhibited a positive relationship with emu spatial abundance, with higher vegetation productivity associated with more favorable habitat suitability. On the other hand, areas with greater fire frequency (higher probability of fire occurrence) indicate lower habitat suitability and lower emu abundance (Fig 3).

**Fig 4:**
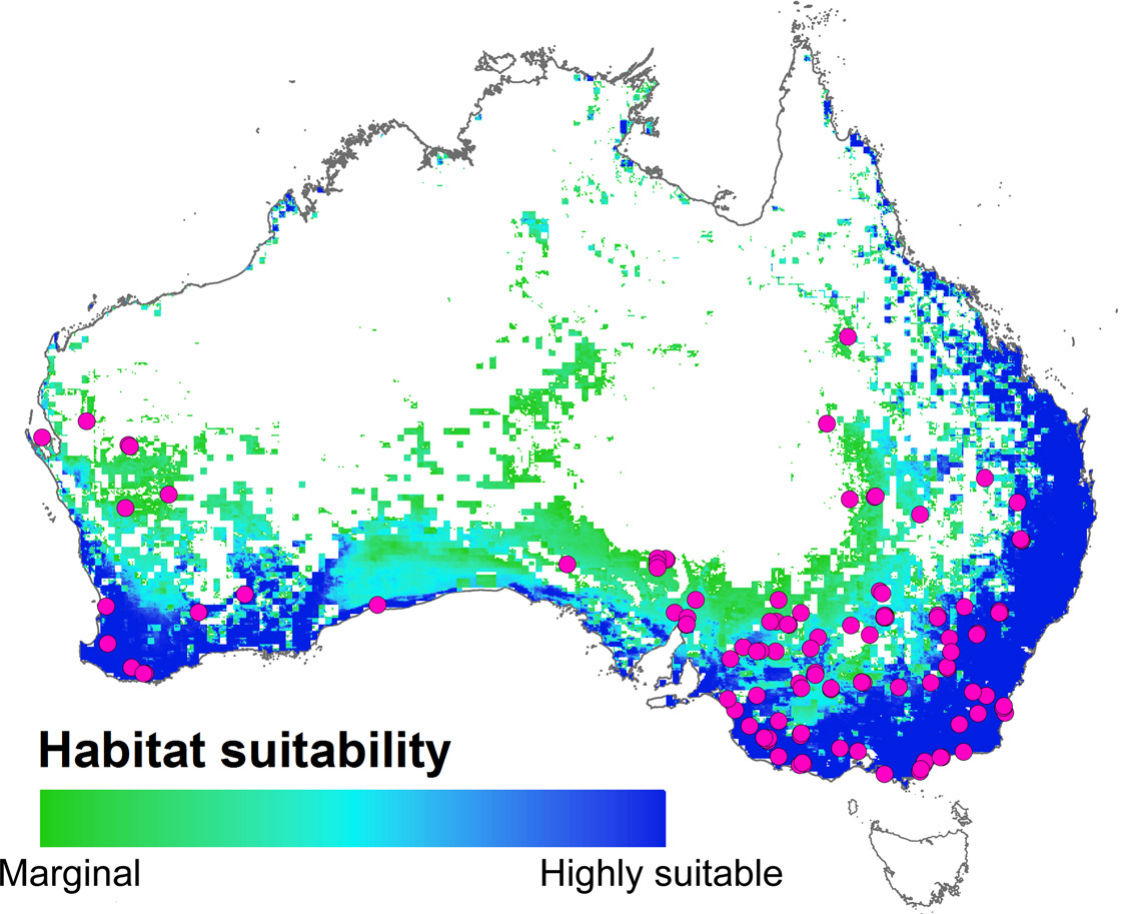
Estimated potential summer habitat suitability map for emu ranging from marginal (60% probability of occurrence) to highly suitable habitats (100%). White areas represent non-suitable habitats (<60% probability of occurrence), while purple circles denote emu summer presence points.

**Fig 5:**
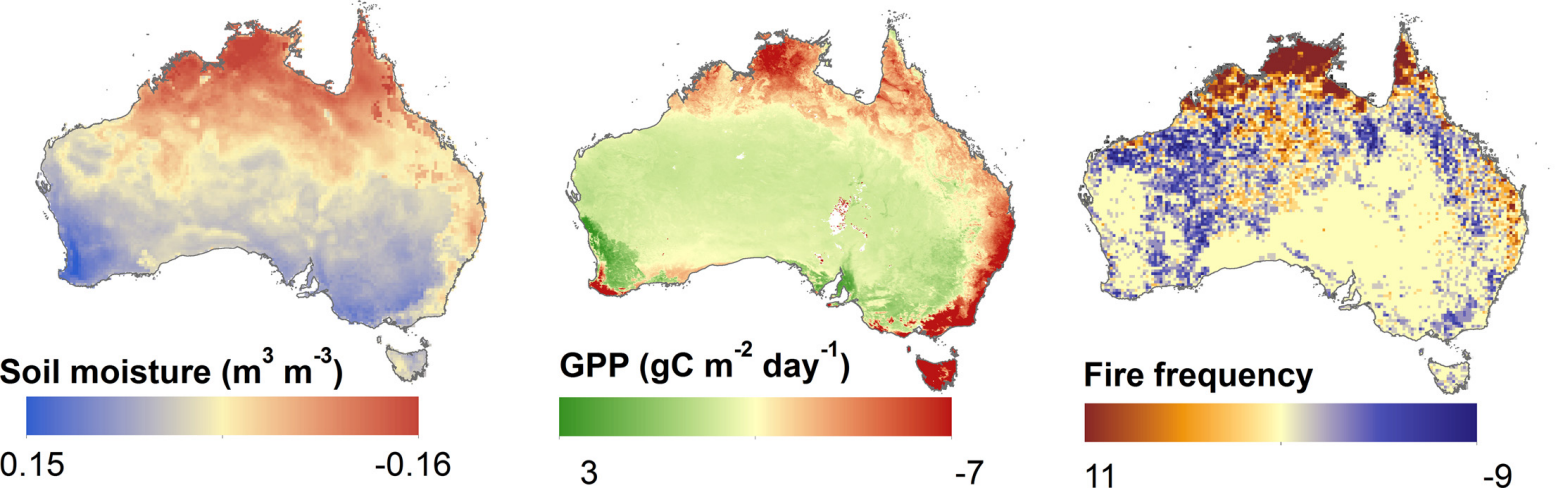
The difference between average winter (July) and austral summer (January) conditions for soil moisture, GPP, and fire frequency (years) defined from the 2000–2010 satellite records. Winter reduction in soil moisture in northern Australia coincides with lower productivity and higher fire frequency in those regions.

The resulting GAM based habitat suitability map (Fig 4) indicates the probability of emu presence, ranging from unsuitable (0% probability of occurrence) to marginal (60%) and highly suitable habitat and certain occurrence (100%); the suitability classification is based on the performance object created by ROCR library in R so that all values below 60% probability of occurrence are considered as areas of absence. These results indicate that the summer range for the emu is limited to southern and eastern parts of Australia that tend to have higher vegetation productivity, lower fire frequency and optimal soil moisture levels (Fig 5).

## Discussion

The results of this study are consistent with previous reports that emus avoid areas with relatively high fire frequency [14]. Greater density of summer emu presence in the southern part of the region can be explained by differences in the environmental variables between summer and winter seasons (Fig 5), which suggests that emus optimize their niche conditions during the dry season by moving into more favorable habitats in southern Australia with greater potential food availability (GPP), lower large wildfire risk and greater water availability. On the other hand, during the summer, northern Australia becomes less suitable as the land is drier with less food availability, and greater fire occurrence.

Species response to fire regimes is difficult to measure because fire is affected by many environmental factors. However, our results indicate that the satellite based fire frequency data, as an indicator of ecosystem susceptibility to fire, is a useful index for representing fire related disturbance effects on emu populations.

A key component of habitat modelling is selecting the most important environmental factors that can provide the most information about species distributions. Most of the studies in the context of species distribution modeling view habitat as a stable part of the ecosystem by selecting average annual values of rather long term climatic variables. However, in Australia and other regions with large seasonal climate variations, many species have migratory dispersal patterns, and seek to avoid environmental restrictions by relocating to more suitable habitats. Many satellite environmental data records are available with potential utility for mapping and monitoring regional habitat suitability and seasonal dynamics, and associated niches for a range of potential species. Many of these data records provide consistent observations and frequent temporal coverage suitable for modeling species niche space dynamics as a result of seasonal movements, which has previously been neglected in species distribution modelling. Selection of environmental variables should be considered in relation to major species niche requirements, including food supply, shelter and available water requirements. Moreover, other satellite data, including vegetation biomass and open water inundation dynamics from satellite microwave remote sensing [50, 51]http://freezethaw.ntsg.umt.edu/, have potential to be used for modeling species seasonal habitat dynamics. However, the relatively coarse spatial resolution of many global satellite data records may limit their utility for species with small scale movement patterns or a narrow niche space. Other than the environmental variables, collecting species locality points that account for dynamic seasonal changes in species migratory distributions can address uncertainties in habitat suitability maps.

One of the limitations of this study was our use of mean seasonal climate attributes and temporally limited emu observational data to define species habitat and movement patterns. Climate variability is dynamic and superimposed on longer-term climate trends that were not addressed in this study. Improved regional monitoring of emu populations and locations would allow for better assessment of how populations may respond to inter-annual climate variability and longer-term climate trends.

The restricted extent of summer suitable habitats for emu has implications for understanding the potential effect of projected climate change on this species. The Millennium Ecosystem Assessment (2005) documents climate change as the largest forthcoming threat to biodiversity across most global biomes. Apart from the direct effect of climate on species, projected regional climate trends could change suitable ranges and migratory patterns for emu and other species[52]. The majority of global climate models represented in the fifth assessment of the Intergovernmental Panel on Climate Change (IPCC) predict warmer and drier conditions for Australian rangelands [53]. Our results indicate that these conditions could promote widespread loss of suitable habitats and niche space for emu, particularly during the austral summer season, with potential restrictions on seasonal habitat availability. Moreover, potential changes in emu habitat and populations could affect the regional ecosystem, as emu play a significant role in seed dispersal for many plant species in Australia [54]. The proposed data and modeling framework can be applied for other regions and species for better understanding and estimation of species occurrence and habitat suitability.

## Supporting Information

S1 File Supporting information including the Pearson correlation values between initial environmental variables (Table A). The Receiver Operator Characteristic (ROC) curve for 10 model runs (Fig A). Partial response and residuals of the GAM fit (Fig B).
(DOCX)
