# Supplementary material for: Remote Sensing Derived Fire Frequency, Soil Moisture and Ecosystem Productivity Explain Regional Movements in Emu over Australia
Source: PLoS One. 2016 Jan 22;11(1):e0147285. doi: 10.1371/journal.pone.0147285 (PMC4723036; doi:10.1371/journal.pone.0147285)
Supplement: S1 File — The Receiver Operator Characteristic (ROC) curve for 10 model runs (Fig A). Partial response and residuals of the GAM fit (Fig B). (DOCX) [file pone.0147285.s001.docx]

Supporting information

Table A. Pearson correlation values between initial environmental variables considered for modeling summer distribution of emu in Australia.

|  | Soil moisture | Rainfall | NDVI | GPP |
| --- | --- | --- | --- | --- |
| Rainfall | 0.69 |  |  |  |
| NDVI | 0.50 | 0.31 |  |  |
| GPP | 0.45 | 0.19 | 0.92 |  |
| Fire frequency | 0.29 | 0.50 | 0.01 | 0.04 |


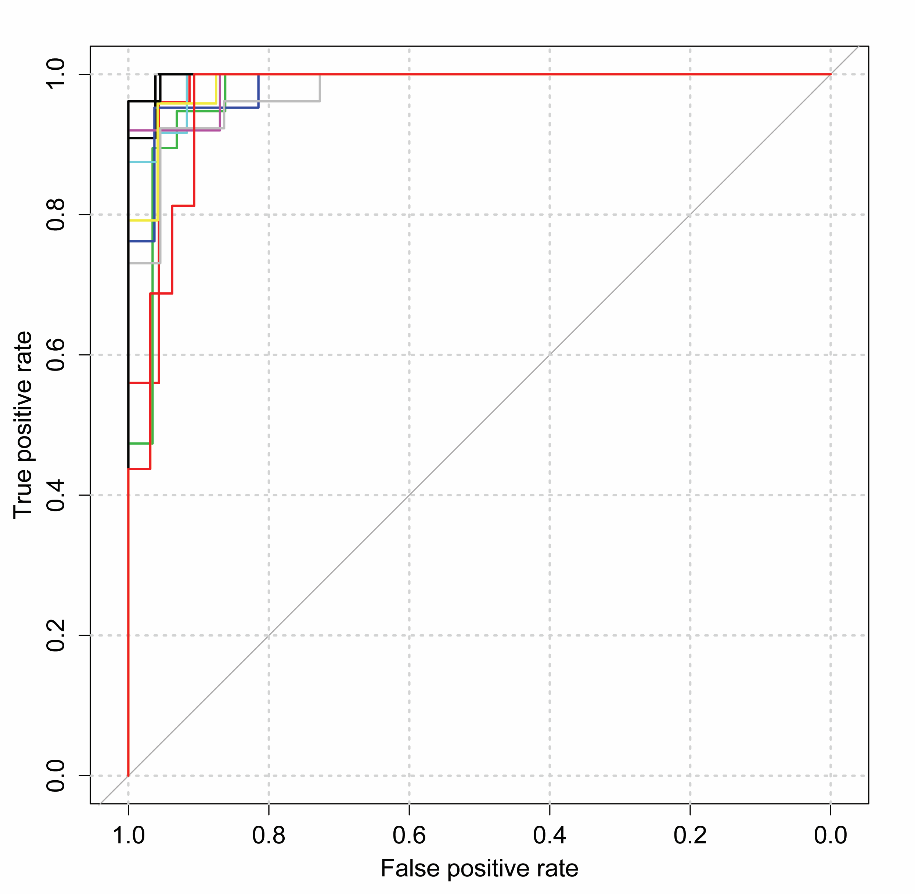


Figure A. The Receiver Operator Characteristic (ROC) curve for 10 model runs resulting from 10-fold cross validation; ROC curves are evaluated on the hold out test data sets and colored according to each run.


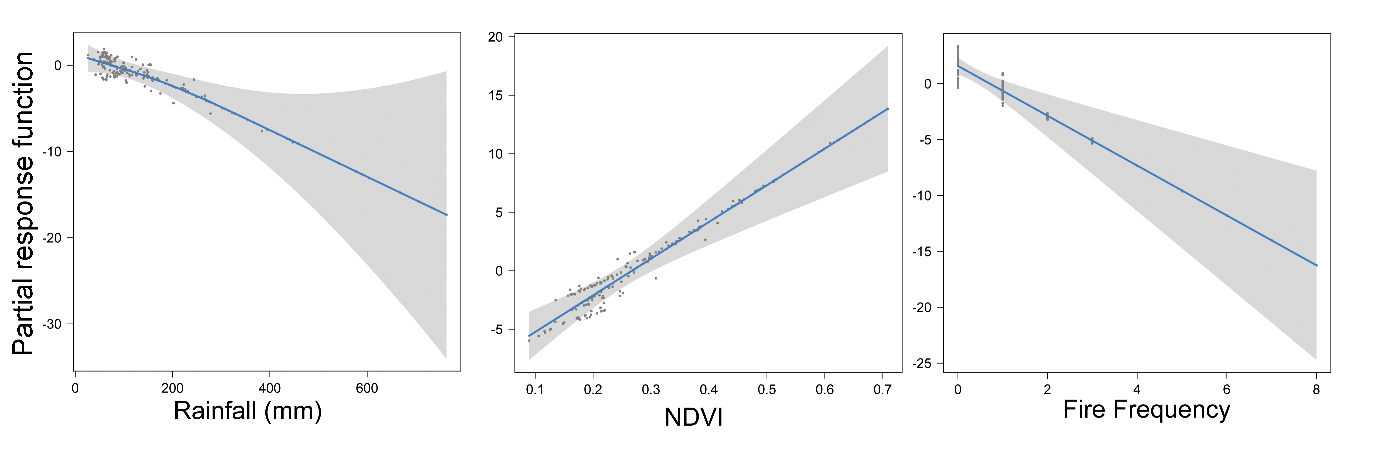


Figure B. Partial response and residuals of the GAM fit using satellite based rainfall, NDVI and fire frequency as explanatory variables. Gray shading defines the confidence intervals associated with the estimates, while the points denote emu presence or pseudo-absence observations. The resulting GAM explained 66 % of the variance in emu observations, which was less than the alternative model employing soil moisture, GPP, and fire frequency inputs.
